# Zinc-Coordination Polymer-Derived Porous Carbon-Supported Stable PtM Electrocatalysts for Methanol Oxidation Reaction

**DOI:** 10.1021/acsomega.0c05843

**Published:** 2021-03-02

**Authors:** Inayat Ali Khan, Amin Badshah, Faiz Ullah Shah, Mohammed A. Assiri, Muhammad Arif Nadeem

**Affiliations:** † Catalysis and Nanomaterials Lab 27, Department of Chemistry, Quaid-i-Azam University, Islamabad 45320, Pakistan; ‡ Chemistry of Interfaces, Luleå University of Technology, SE-97187 Luleå, Sweden; § Department of Chemistry, Faculty of Science, King Khalid University, P.O. Box 9004, Abha 61413, Saudi Arabia

## Abstract

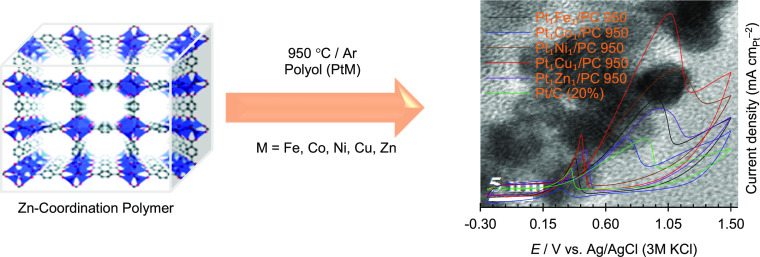

Porous carbon (PC) is obtained by carbonizing a zinc-coordination polymer (MOF-5) at 950 °C and PtM (M = Fe, Co, Ni, Cu, Zn) nanoparticles (NPs), which are deposited on PC using the polyol method. Structural and morphological characterizations of the synthesized materials are carried out by powder X-ray diffraction (PXRD), X-ray photoelectron spectroscopy (XPS), and high-resolution transmission electron microscopy (HRTEM), and the porosity was determined using a N_2_ adsorption/desorption technique. The results revealed that PtM NPs are alloyed in the *fcc* phase and are well dispersed on the surface of PC. The electrochemical results show that PtM/PC 950 catalysts have higher methanol oxidation reaction (MOR) performances than commercial Pt/C (20%) catalysts. After 3000 s of chronoamperometry (CA) test, the MOR performances decreased in the order of Pt_1_Cu_1_/PC 950 > Pt_1_Ni_1_/PC 950 > Pt_1_Fe_1_/PC 950 > Pt_1_Zn_1_/PC 950 > Pt_1_Co_1_/PC 950. The high MOR activities of the synthesized catalysts are attributed to the effect of M on methanol dissociative chemisorption and improved tolerance of Pt against CO poisoning. The high specific surface area and porosity of the carbon support have an additional effect in boosting the MOR activities. Screening of the first row transition metals (*d*^5+*n*^, *n* = 1, 2, 3, 4, 5) alloyed with Pt binary catalysts for MOR shows that Pt with *d*^8^ (Ni) and *d*^9^ (Cu) transition metals, in equivalent atomic ratios, are good anode catalysts for alcohol fuel cells.

## Introduction

Alcohol fuel cells (FCs) have been considered as competent and green energy technology for the future owing to their high-energy output, low waste release, and hygienic operation.^1−3^ Among the different types of FCs, direct methanol fuel cells (DMFCs) utilizing methanol as a fuel have been extensively studied in the last 20 years due to the advantages of room temperature liquid fuel, which is less explosive and can be easily stored. The efficiency of DMFCs is dependent on the electrode catalyst (platinum) composition, stability, and durability.^1−3^ One of the major problems with the platinum electrodes is the CO-poisoning during fuel oxidation.^4^ To overcome this problem, several binary catalysts such as PtFe,^5,6^ PtCo,^6−8^ PtNi,^6,9^ PtCu,^10,11^ PtZn,^12,13^ PtAg,^14^ and PtSn^15^ have been electrochemically tested for fuel cell reactions. The improved catalysis was attributed to the bifunctional mechanism of fuel oxidation.^4^ Further, a cost decrease of FCs has also been highlighted in the studies of PtM catalysts.^5−15^ All the PtM binary catalysts have presented optimum performance of fuel oxidation and good CO tolerance. However, a systematic and comparative study about PtM catalysts (M = transition metals having *d*^5+*n*^ electrons) on an alcohol oxidation reaction is rarely found in the literature.

The structure and morphology of PtM NPs have an important role in their catalytic performance.^3,16^ Previously, alloy nanowires, alloy nanotubes, nanoflowers, and core–shell nanostructures have been synthesized and studied for methanol oxidation reaction. Alloy nanowires and nanotubes have shown higher activity than nanospheres because the nanospheres are easily subjected to oxidation and dissolution.^17,18^ Three-dimensional nanostructures have often presented higher catalytic performance than the one-dimensional nanostructures.^19,20^ The platinum group metals (PGM) in a 1:1 ratio composition supported on reduce graphene oxide (rGR-PtPd) is reported for methanol oxidation reaction. In the electrochemical tests, the PtPd catalysts on rGO have presented high MOR activities than homemade Pt/rGO, Pd/rGO, and commercial Pt/C catalysts. The high electrochemical performance of the synthesized catalysts was attributed to its large electrochemical surface area, ultrafine particles size, and synergetic effect between the alloyed metals.^21,22^ Chen et al. have reported nanoflower-like Pd@Pt supported on graphene, which showed higher performance than PdPt nanofoams for MOR.^23^ Similarly, Tan et al. reported Au@Pd core–shell nanoparticles of higher MOR catalytic activity than Au free Pd nanoparticles.^24^ AuPd alloy networks have been reported for ethanol electroxidation, and the importance of catalyst composition for the catalytic performance was also highlighted.^25^

Various catalytic supports such as polyindol-functionalized CNTs, sulfur-doped CNTs, and Fe-, N-, and S-tridoped graphene have been extensively explored for MOR where the support structure and functionalization played a crucial role in both catalytic activity and durability.^26−28^ An efficient carbon support should have a high surface area that can ensure uniform particle size distribution, electronic good conductance and high corrosion resistance, strong cohesive force to metal particles, and easy formation of metal nanoparticles on its surfaces. The current state-of-the-art catalyst is Pt (Pt-alloyed) supported on porous conductive carbon of surface area > 100 m^2^ g^–1^.^29−33^ The major drawback of these carbon materials is the corrosion caused by the electrochemical reactions during the operating environment of fuel cells, which limits the catalyst durability and reliability. The corrosion effect is severe when the fuel cell is operating at high temperatures that lead to degradation of the catalytic material. For instance, Wilkinson et al.^34^ and Zhang et al.^35^ reported the oxidative degradation of the carbon support at high temperatures when the fuel cell was operated for more than 500 h. The corrosion resulted to a loss in the electrocatalytic surface area and Pt particle separation from the support surface or agglomeration to larger size. In this regard, the development of more durable catalysts and carbon support materials is needed especially for fuel cells operating at a high temperature of >90 °C. Kim et al. have prepared high surface area graphene-like 3D microporous carbons (3D CMs) from ethylene gas in the cages of lanthanum-exchanged zeolite at high temperature.^36^ The resultant carbon has an electrical conductivity two order of magnitude higher than that of amorphous mesoporous carbon. The same method of carbon synthesis was also adopted for the large-scale carbon synthesis in different topologies corresponding to the different pore sizes of zeolite templates. Tüysüz and co-workers also used the same synthetic method for three-dimensional-ordered microporous carbon materials, and the most promising sample of cobalt–carbon was treated with NH_3_ for nitrogen doping.^37^ The nitrogen doped cobalt–carbon sample was used as an oxygen-reducing fuel cell catalyst, which demonstrated good electrochemical stability and almost similar activity to the commercial Pt/C 20% catalyst. Herein, we prepared high surface area porous carbon (PC) from self-sacrificed zinc-coordination polymer (MOF-5 as a soft template) under inert carbonization in a single step without the acid-leach out process of template.^9^ After structural and morphological characterization of the PC, a series of PtM (M = Fe, Co, Ni, Cu, Zn) nanoparticles were prepared and deposited on the surface of carbon using the polyol synthesis method. The synthesized catalysts were evaluated for methanol oxidation reaction, and as a result, PtCu and PtNi bimetallic combinations supported on PC exhibited high catalytic activities and long-term stability. The high catalytic activities can be attributed to the appropriate metal composition, their mutual electron interaction, and alloying in one stable crystalline phase that was further boosted by the high surface area and durable porous carbon support. It is anticipated that this method of PC and PtM integration will offer a simple and convenient way to design a variety of highly efficient and low-cost catalysts for direct methanol fuel cells.

## Results and Discussion

### Structural and Morphological Characterization

**Figure 1:**
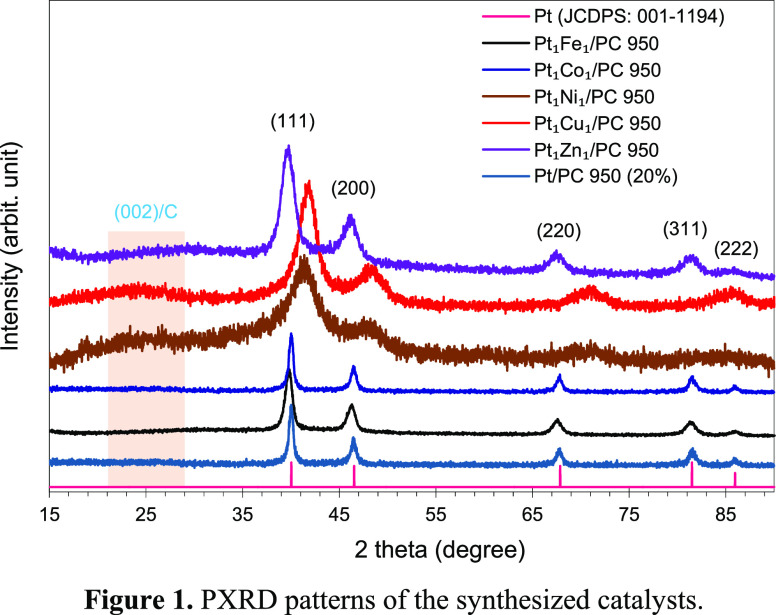
PXRD patterns of the synthesized catalysts.

The PXRD pattern-a (Figure S1) of the synthesized coordination polymer is well matched with the simulated pattern of MOF-5,^38^ while the PXRD pattern-b have peaks at 2θ = 25 and 45° representing the (002) and (100) crystallographic planes of carbon. The PXRD patterns of the catalysts together with the *fcc*-Pt reference pattern (JCPDS: 001-1194) are presented in Figure 1. In the PXRD pattern of Pt/PC 950 (20%) and Pt_1_Co_1_/PC 950 catalysts, peaks at 2θ = 40.1, 46.5, 67.9, 81.6, and 86.1° are due to the (111), (200), (220), (311), and (222) lattice planes of face-centered cubic-Pt, respectively. In the case of Pt_1_Fe_1_/PC 950 and Pt_1_Zn_1_/PC 950, the corresponding diffraction peaks are negatively shifted to 39.6° for (111), to 46.3° for (200), to 67.6° for (220), to 81.5° for (311), and to 85.8° for (222) lattice planes. Compared to the Pt reference pattern, the PXRD pattern of Pt_1_Ni_1_/PC 950 composition is shifted to 2θ = 41.3, 48.2, 71.1, and 86.1° representing the (111), (200), (220), and (222) lattice planes, respectively. Similarly in the case of the Pt_1_Cu_1_/PC 950 catalyst, the PXRD peaks are shifted to higher 2θ values of 41.9, 48.5, 71.2, and 86.1° corresponding to the (111), (200), (220), and (222) lattice planes of Pt, respectively. In comparison to the reference peaks of *fcc*-Pt, a positive or a negative shift in peaks of the PtM catalysts confirms alloy formation.^39^ The PXRD analysis clearly show the crystalline (for Pt, PtFe, and PtCo) and semi-crystalline (for PtNi, PtCu, and PtZn having comparatively broader peaks) natures of the catalysts synthesized by the polyol method. A broad peak associated to the carbon (002) lattice plane at 2θ = 25° is not observed in the patterns of Pt, PtFe, and PtCo due to the crystalline nature of the alloyed nanoparticles (NPs), which produced low width and high intensity diffraction peaks of PtM NPs. The carbon (002) peak is comparatively more obvious in the case of PtNi, PtCu, and PtZn probably due to the semi-crystalline nature of the alloyed NPs, which produced comparatively high-width and low-intensity diffraction peaks.

**Figure 2:**
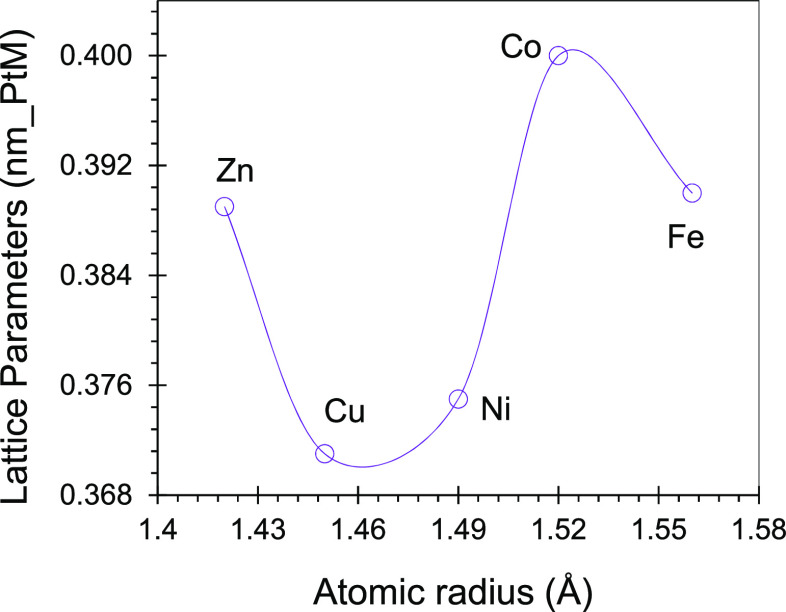
Dependence of lattice parameters on the atomic radii of *d*^5+n^ transition metals.

The diffraction peaks (Table S1) of the Pt_1_M_1_ catalysts are shifted toward larger (for PtCo, PtNi, and PtCu) or smaller (for PtFe and PtZn; very small shift) 2θ angles in comparison to the Pt peaks in the reference pattern (Figure 1 and Figure S2). The shift in the 2θ position is due to the substitution of smaller atoms (Fe = 1.56 Å, Co = 1.52 Å, Ni = 1.49 Å, Cu = 1.45 Å, and Zn = 1.42 Å)^40^ into the Pt lattice (1.77 Å),^40^ which decreased the overall unit-cell dimension of the platinum alloy.^41,42^ The decreasing order of positive difference between the 2θ_PtM_ and 2θ_Pt-reference_ at (111) decreased in the order of Pt_1_Cu_1_ > Pt_1_Ni_1_ > Pt_1_Co_1_ > Pt_1_Fe_1_ > Pt_1_Zn_1_, showing good alloying characteristics of the first-row transition metals with Pt (Figure S2 and Table S1). The lattice parameters (LP) of the synthesized PtM/PC 950 catalysts (Table S1) are different from that of standard Pt (0.392 nm) and that of Pt (20%) on the PC 950 catalyst (0.388 nm) exhibiting alloy formation. The dependence of the Pt unit cell parameter on the atomic radii of dopant metals was depicted in Figure 2. It can be seen that the lattice parameter of the PtM catalyst increases with the atomic radius from Cu through Ni to Co, while it decreases from Zn to Cu and from Co to Fe, presenting a wave curve. The high lattice parameter value of PtM was calculated (0.400 nm, Table S1) when Pt was alloyed with Co, and the lowest lattice parameter value of PtM was calculated (0.371 nm, Table S1) when Pt was alloyed with Cu. It is noteworthy that during electrochemical evaluation of the catalysts for methanol oxidation reaction, Pt_1_Co_1_/PC 950 was the worst, while Pt_1_Cu_1_/PC 950 was the best catalyst among the options. The following Scherrer eq 1 was used to calculate the average crystallite sizes (*D*_cryst_):
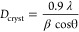
1where *D*_cryst_ is the diameter of the crystallite size (nm), λ is the wavelength (0.15406 nm) of X-ray from Cu Kα, θ is the Bragg angle, and β is the full width at half-maximum (FWHM). The calculated values of *D*_cryst_ are given in Table S1. From the PXRD, the catalysts can be ordered with increasing *D*_cryst_ as Pt_1_Cu_1_ ≈ Pt_1_Ni_1_ < Pt_1_Fe_1_ ≈ Pt_1_Zn_1_ ≪ Pt_1_Co_1_. The crystallite size of PtCo (about 24 nm) is 4 times larger than PtCu and PtNi and 1.5 times larger than PtFe and PtZn, although all the catalysts were prepared using the polyol method. The high crystallite size of the PtCo catalyst is probably due to the high tendency of PtCo nonmetric particles to aggregate. Therefore, the synthesis of PtM (M = Fe, Co, Ni, Cu, Zn) catalysts in uniform particle size by one method is not possible.

The experimental metal loading of all the catalysts is close to the theoretical metal loading calculated from the salts. Calculation of the metal amount was performed according to Vegard’s law assuming PtM as solid solutions. The PtCu and PtNi were shown to form a solid solution, with a composition of approximately Pt_13_Cu_11_ and Pt_15_Ni_12_, respectively, which is very close to the values from the ICP-OES analysis. However, the calculated percent amount of M (Fe, Co, Zn) is significantly lower than the percent amount of Pt (Table S1). There are some reports where they observed that the non-noble metal part of the alloying component in PtM-type catalysts could be found in the amorphous form that cannot be detected by the powder X-ray technique.^42,43^

**Figure 3:**
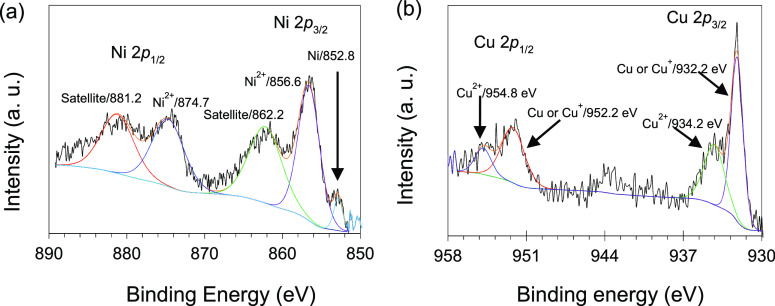
Deconvoluted XPS spectra of the Ni 2p core line (a) and Cu 2p core line (b).

The XPS survey spectra presented in Figure S3 have peaks for C, O, Pt, Ni, and Cu. The C 1s and O 1s core lines were fitted to sub-peaks at 284.5 eV for sp^3^/sp^2^ carbon, peaks at 285.2 and 286.1 eV (in C 1s), while the peak at 533.6 eV (in O 1s) were attributed to the C–O and C=O functional groups, respectively (Figure S4a,b). The O 1s core line sub-peak at 532.0 eV can be assign to the oxygen bonded to metal.^44^ The Pt XPS core lines in Pt_1_Ni_1_/PC 950 and Pt_1_Cu_1_/PC 950 were fitted to the pair of triplets corresponding to lower (4*f*_7/2_) and higher (4*f*_5/2_) binding energies, Figure S4c,d. In each triplet, the lower binding energy peaks in the range of 71.1–71.4 and 74.5–74.7 eV are due to elemental Pt, the middle binding energy peaks in the range of 72.0–72.4 and 75.6–75.8 eV are due to PtO, and the high energy binding peaks at 73.0 and at 77.3 eV are due to PtO_2_.^44,45^ The observed binding energies of Pt are negative shifted in comparison to the reference Pt binding energy value (71.8 eV) due to electron interaction of Pt with transition metals.^46^ The electronegativity of Pt (2.28) is higher than Ni (1.80) and Cu (1.90), and the electron density would be more toward Pt. Such electron interaction can modify the *d*-band of Pt, which is significant for catalytic performance and stability.^47−49^ A porous carbon support with surface organic functional groups was found interactive with Pt, and the catalyst produced high methanol oxidation performance.^47^ Herein, the PC 950 carbon support also interacted with PtM NPs and resulted to promote a change in the Pt binding energy value. The fitted core-line of Ni 2p is presented in Figure 3a. In the deconvoluted spectrum, the Ni^2+^ peaks at 856.6 and 874.7 eV along with broad shake-up satellite peaks at 862.2 and 881.2 eV were observed. The metallic state of nickel was also detected at 852.8 eV associated to Ni.^50^ From the XPS analysis, it is obvious that the proportion of metallic Ni on the surface of NPs is very low and the surface is nickel oxide rich, probably due to surface oxidation, along with some metallic nickel. In the fitted core-line spectra of Cu 2p, the strong peaks at 932.2 eV (in 2p_3/2_) and 952.2 eV (in 2p_1/2_) are assigned to Cu/Cu^+^. Peak pairs at 934.2 eV (in 2p_3/2_) and 954.8 eV (in 2p_1/2_) are assigned to Cu^2+^ (Figure 3b). The XPS of the PtCu catalyst show that metallic copper is the main component on the NP surfaces.^45,51^ The Cu^2+^ satellite peak at about 944 eV in the Cu 2p spectrum is also observed (Figure 3b). There is a slight shift in the binding energy of both Ni and Cu as compared to the bulk Ni (852.6 eV) and Cu (932.4 eV) values showing electron interaction with Pt, resulting to alloy formation. From the XPS analysis, the composition of Pt_1_Ni_1_/PC 950 is 75.17 wt % C, 7.51 wt % O, 11.01 wt % Pt, and 4.21 wt % Ni while that of Pt_1_Cu_1_/PC 950 is 84.83 wt % C, 6.33 wt % O, 4.31 wt % Pt, and 3.74 wt % Cu (Table S2). In comparison to the ICP-OES results (Table S1), the low percentage of Ni and Cu detected in XPS is probably due to their substrate nature in the NP composition^46^ and due to the XPS surface detection property.

In the gas adsorption/desorption analysis, the PC 950 have presented a Type IV isotherm (Figure S5a), which is typical for porous materials of micro- and mesopore regions. During measurements, a sharp increase in the gas adsorption at low relative pressure is due to the presence of micropores (less than 2 nm), the adsorption/desorption hysteresis at medium relative pressure is due to mesopores (2–50 nm) and again increase in gas adsorption at high relative pressure is due to macropores (>50 nm).^52,53^ The porosity generated in the carbon is due to gases and zinc metal evaporation during carbonization of the coordination polymer.^54^ The PC 950 has three pore regions and can be a good option as support for PtM that could promote molecule mass diffusion and catalyst stability. The PtNi and PtCu NPs have decreased N_2_ adsorbed volume by manyfold (Figure S5a) and consequently the surface area and porosity decreased (Table 1).^55^ The pore region of PC 950 is also reflected in the pore size distribution plot (Figure S5b) that has volume adsorption peaks at 0.4, 1.3, and at 1.5–3.3 nm. The volume adsorption peak at 0.4 nm was drastically decreased by the addition of PtNi and PtCu NPs. This decrease in surface area and porosity of the catalysts can be explained by the sinking of an important portion of metal NPs into the micropore region (<2 nm) of the carbon support^56^ that resulted into a drastic decrease in N_2_ adsorbed volume and surface area. This is also an indication that PtM NPs are distributed in the micropores of PC 950 that is obviously observed in TEM images.

**Table 1:** Surface Parameters of the Present Work Carbon, Catalysts, and Literature-Reported Carbons

carbons/catalysts	BET (m^2^ g^–1^)	pore volume (cm^3^ g^–1^)	pore size (nm)	ref
Vulcan XC72	235	0.67		(57)
CMK-8-I	1060	1.26	4.9	(58)
CMK-8-II	1149	1.48	3.2	(58)
MDC-1	3174	4.06		(59)
MC	1812	2.87		(60)
MPC 950	1455	2.03		(61)
PC 950	1453	2.00		this work
Pt_1_Ni_1_/PC 950	234	0.89		this work
Pt_1_Cu_1_/PC 950	231	0.33		this work

**Figure 4:**
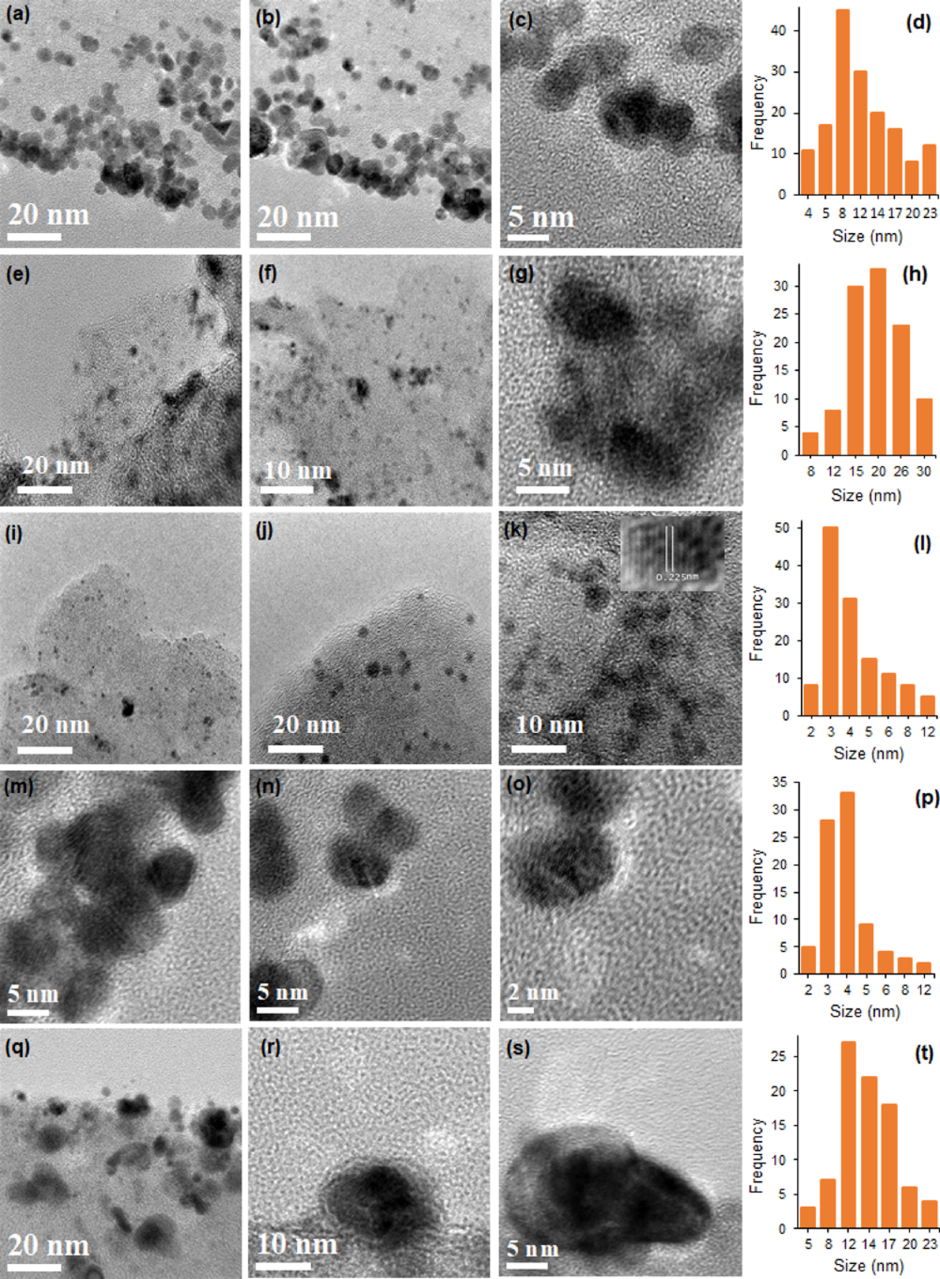
TEM images and histograms of Pt_1_Fe_1_/PC 950 (a–d), Pt_1_Co_1_/PC 950 (e–h), Pt_1_Ni_1_/PC 950 (i–l), Pt_1_Cu_1_/PC 950 (m–p), and Pt_1_Zn_1_/PC 950 (q–t).

The TEM images (Figure 4) show spherical, dispersed, and carbon surface-embedded metal NPs. As can be seen in Figure 4i, the particle size of carbon is larger than 50 nm supporting agglomerated metal NPs. These agglomerated metal NPs are embedded in the carbon matrix, which can stabilize them from leaching in acid solution.^41^ The inset of Figure 4k is a high resolution image of a single NP, displaying interwoven lattice fringes (0.225 nm) of the Pt_1_Ni_1_ alloy corresponding to the (111) plane. The observed lattice plane value is smaller than the standard reference value of Pt (0.227 nm),^55^ which means alloy formation and electron interaction of Pt and Ni. The histogram of PtNi (Figure 4l) and PtCu (Figure 4p) shows a narrow particle-size distribution, while the histogram of PtFe (Figure 4d), PtCo (Figure 4h), and PtZn (Figure 4t) shows a relatively broad particle-size distribution. In the case of PtNi (Figure 4l) and PtCu (Figure 4p), the average particle sizes are approximately 3 and 3.2 nm, respectively. These results are not coincided with that of PXRD due to difference between the particle size and crystallite size. Similarly, Pt_1_Fe_1_/PC 950, Pt_1_Co_1_/PC 950, and Pt_1_Zn_1_/PC 950 catalysts have average particle sizes of 9.41, 23, and 13.52 nm, respectively. PtNi and PtCu alloys have a narrower distribution and smaller particle size than that of the PtFe, PtCo, and PtZn alloys. The small particle sizes of Pt_1_Ni_1_/PC 950 and Pt_1_Cu_1_/PC 950 is beneficial for the improved catalytic activities toward MOR.

### Electrocatalytic Activities of PtM/PC 950 Catalysts

**Figure 5:**
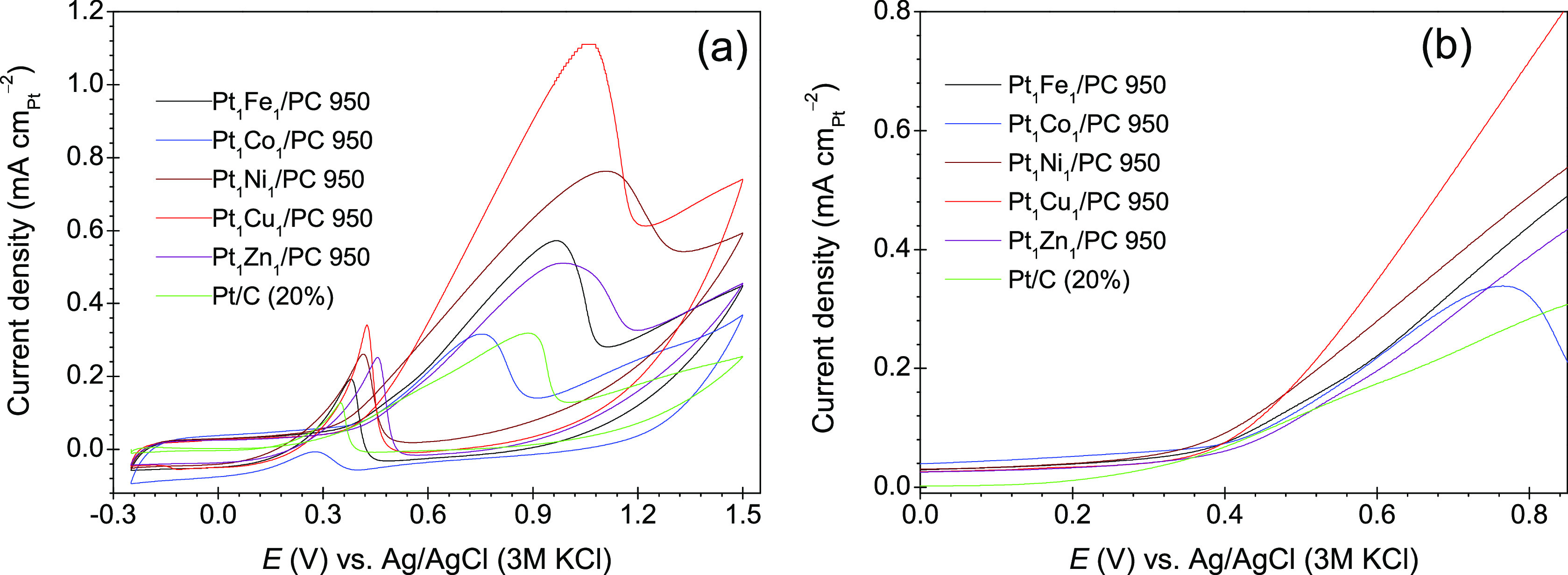
CV curves (a) and LSV curves (b) of the synthesized and commercial catalysts. The electrolyte solution was HClO_4_ (0.1 mol L^–1^) having CH_3_OH (1 mol L^–1^).

The cyclic voltammetry (CV) and the linear sweep voltammetry (LSV) responses of the catalysts for methanol oxidation reaction (MOR) are presented in Figures S6 and S7 and Figure 5. There are hydrogen adsorption/desorption peaks below 0.07 V versus Ag/AgCl and Pt oxidation/reduction peaks at 0.15–0.71 V versus Ag/AgCl (Figure S6). The electrochemical surface areas (ECSA) were calculated from the charge accommodated under the hydrogen desorption peak using the following eq 2:^62^
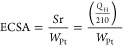
2where *S*_r_ is the real surface area of catalyst (cm_Pt_^2^), *Q*_H_ is the total charge under hydrogen desorption curve (μC), *W*_Pt_ is the Pt loading (g cm^–2^) on the electrode surface and 210 is the charge (μC cm_Pt_^–2^) required for hydrogen oxidation on a shiny Pt surface.^62^ The synthesized catalysts have comparatively low ECSA than the commercial catalyst (20% Pt on Vulcan X72, Table 2) probably due to 15% Pt loading and carbon layers encapsulation of the PtM NPs. A decreasing order of the PtM catalysts based on calculated ECSA is Pt_1_Ni_1_ (78.40 m^2^ g^−1^) > Pt_1_Cu_1_ (63.20 m^2^ g^−1^) > Pt_1_Zn_1_ (52.30 m^2^ g^−1^) > Pt_1_Fe_1_ (50.60 m^2^ g^−1^) > Pt_1_Co_1_ (42.32 m^2^ g^−1^). A possible reason for the lower ECSA of Pt_1_Cu_1_/PC 950 than Pt_1_Ni_1_/PC 950 is the carbon layering of the PtCu NP surfaces. In cyclic voltammetry, for MOR, when the potential was scanned from −0.25 to +1.5 V, all the samples displayed two characteristics peaks of methanol oxidation (Figure 5a). One oxidation peak is in the potential range of 0.63–1.19 V for the deprotonation of methanol, and another oxidation peak is in the potential range of 0.19–0.51 V for the oxidation of adsorbed carbon monoxide.^63^ The efficiency of a catalyst can be judged based on the following parameters: (a) anodic peak current density (*I*_ap_) at respective anodic potential (*E*_ap_), (b) onset potential (*E*_onset_), showing methanol oxidation activity, and (c) the forward to reverse peak current density (*I*_f_/*I*_r_) ratios, reflecting the catalyst tolerance.^64^ As can be seen in Table 2 and Table S3, the *I*_ap_ of the synthesized catalysts toward MOR is in the order of Pt_1_Cu_1_ > Pt_1_Ni_1_ > Pt_1_Fe_1_ > Pt_1_Zn_1_ > Pt_1_Co_1_. The catalysts Pt_1_Cu_1_/PC 950, Pt_1_Ni_1_/PC 950, Pt_1_Fe_1_/PC 950 and Pt_1_Zn_1_/PC 950 have higher *I*_ap_ values, while Pt_1_Co_1_/PC 950 have comparable *I*_ap_ values to those of the commercial Pt/C 20% catalyst (0.33 mA cm_Pt_^−2^). The forward anodic peak current density of MOR on Pt_1_Cu_1_/PC 950 is 2.00 times larger than that of Pt_1_Co_1_/PC 950. The onset potentials (from the low potential part of linear sweep voltammetry, Figure 5b) of the synthesized catalysts are more negative than commercial catalyst, which means that the PtM are more active in catalyzing methanol molecules than Pt/C (20%). The *I*_f_/*I*_r_ ratios are 2.97, 3.00, 3.38, and 2.04 for PtFe, PtNi, PtCu, and PtZn, respectively, which are larger than that of Pt/C (20%), showing comparatively high CO tolerance of the synthesized catalysts. The electrochemical data of the PtM catalysts supported on PC 950 carbon clearly revealed that the addition of *d*^5+*n*^ (*n* = 1, 3, 4, 5) electron transition metals (Fe, Ni, Cu, Zn) is promising for increasing the MOR specific activity over Pt-binary catalysts. In comparison to the commercial and to the other options, the PtCu- and PtNi-based catalysts have exhibited high MOR performances as they have comparatively high *I*_ap_ and *I*_f_/*I*_r_ ratios.

**Table 2:** Electrochemical Parameters of the Literature Reported and Synthesized Catalysts

catalysts	ECSA (m^2^ g^–1^)	*I*_ap_ (mA cm_Pt_^–2^)	*J*_m_ (A mg_Pt_^–1^)	ref
Pt_84_Fe_16_ UNWs	48.1	0.96	0.46	(6)
Pt-Co NF	50.0	8.56	4.28	(7)
PtCu_3_-CTAC/C			0.51	(11)
PtCu_3_-CTAB/C			0.38	(11)
10%-PtZn@NC-800		3.30	1.02	(13)
PtAgNTs/C	66.7		0.73	(14)
PtCo ERD NCs/C	35.0	6.80	1.20	(16)
PtNi/NC	58.0	0.60	1.50	(31)
A-Pt_14_Cu_86_	118.6	9.26	0.42	(42)
Pt_1_Cu_0.25_ ANCs/MWCNT	70.9		1.24	(44)
Pt_1_Cu_1_Co_1_Ni_1_	43.8		0.45	(45)
PdNi@Pt CSNSs	32.5		0.96	(46)
Pt-Co NWs (15 h)	46.4	5.57	2.58	(62)
PtZn/MWNT-E (3.2 nm)		1.03		(71)
Pt_1_Ni_1_/PC 950	78.40	0.76	0.41	this work
Pt_1_Cu_1_/PC 950	63.20	1.14	0.47	this work

The high electrochemical performances of the synthesized catalysts toward MOR is attributed to the appropriate metal composition in alloy in which the transition metals can facilitate the electron transfer from M (Fe, Co, Ni, Cu, and Zn) to Pt while decreasing Pt dissolution and agglomeration in an acid medium. It has also been observed in the literature that Cu-modified electronic and geometric structures of Pt resulted to high electrocatalytic activity and decreased CO poisoning.^65^ The electrochemical deposition of Pt–Cu, Pt–Ni, and Pt–Co on the glassy carbon electrode for methanol oxidation have been reported by Sotiropoulos and co-workers.^66^ Similar to the present study, Pt(Cu) have exhibited enhanced catalytic activity toward methanol oxidation in both variable potential and in constant potential experiments. The high catalytic activity was explained in terms of the effect of Cu, Ni, and Co on methanol oxidation and decreasing CO poisoning at Pt surfaces. Nørskov and co-workers explained the effect of 3d transition metals on the electronic and surface properties of Pt (sub-surface) using the density functional theory.^67,68^ They observed that the Pt surface *d*-band was broadened and become lowered in energy by interaction with the 3d metals and resulted in weaker dissociative adsorption energies of hydrogen and oxygen on these surfaces. This “*d*-band center modification theory” can predict the affinity of transition metals (*d*^5+n^) toward O, CO, H, *etc*., adsorbates. The lower the center lies, the lower is the affinity of the metals to the intermediates. Nørskov and co-workers observed that the presence of transition metals broadened the Pt *d*-band with the effect increasing from Cu (*d*^5+4^) to Fe (*d*^5+1^), which in turn decreases the adsorption tendency of Pt.^69^ Therefore, CH_3_OH and intermediate CO (Pt poison) adsorptions are predicted to be decreased on the PtM (M = Fe, Co, Ni, Cu, Zn) alloy catalysts. It seems that Pt_1_Cu_1_ and Pt_1_Ni_1_ catalysts are considered by a reasonable change in the *d*-band that decreased the CO adsorption to a favorable level without lowering methanol chemisorption. On the other hand, a considerable change in the *d*-band structure of Pt with Co resulted in an unacceptable low degree of methanol chemisorption and low MOR.^66^ Xu et al. reported a nanoporous PtFe alloy for MOR, which consists of interconnected ligaments.^70^ The as-synthesized catalysts exhibited high MOR activity, durability, and CO tolerance in comparison to the nanoporous Pt and commercial Pt/C catalysts. The theoretical (DFT) and experimental (XPS) observations revealed that alloying of Fe with Pt resulted in a *d*-band downshift, leading to improved MOR activity and stability in a long-term potential scanning test under low pH. Using mesoporous silica template, PtZn intermetallic NPs up to 3.2 ± 0.4 nm decorated on multiwalled carbon nanotubes for methanol oxidation have been reported.^71^ The PtZn NPs exhibited about 10 times higher MOR performance in both acidic and basic media in comparison to PtZn NPs synthesized without mesoporous silica. Through DFT evaluation of the PtZn system, the high MOR activity was attributed to the small size, high density of corner active sites, low energy pathway of non-CO, and generation of a stable OH* intermediate on Zn. In the present studies, PtFe and PtZn have higher specific activities than commercial Pt/C catalysts probably due to the *d*-band structure modification of Pt and low energy path of MOR.

**Figure 6:**
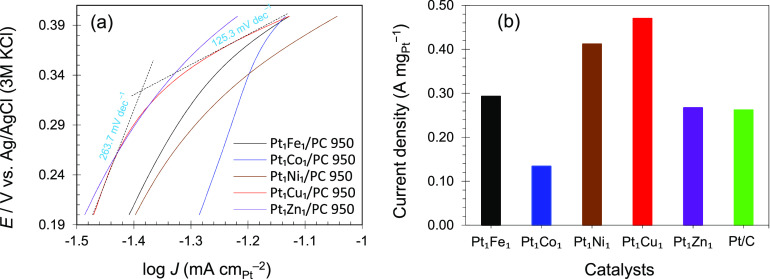
Tafel plots (a) and mass activity plots (b) of the synthesized catalysts. The electrolyte solution was HClO_4_ (0.1 mol L^–1^) having CH_3_OH (1 mol L^–1^).

According to the kinetic theory, the rate-determining step in the electrochemical reaction is the first electron transfer with a Tafel slope of 118 mV dec^–1^.^72^ The Pt_1_Cu_1_/PC 950 (125 mV dec^–1^) and Pt_1_Ni_1_/PC 950 (132 mV dec^–1^) produced almost similar Tafel slopes (Figure 6a) in the potential range of 0.1–0.26 and 0.33–0.4 V, which means that the rate determining step is the first CH bond splitting of methanol. MOR is a complex reaction that involve adsorption, electrons transfer, adsorption, and oxidation of CO, and the mechanism of methanol oxidation on PtM type catalysts can be presented as^73^
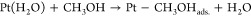
3
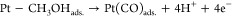
4
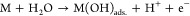
5
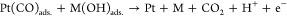
6

The Tafel slopes of Pt_1_Cu_1_ and Pt_1_Ni_1_ are close to the kinetic theory value, which means that eq 4 is the rate-determining step. The methanol dehydrogenation reaction (equation 4) completes in many fundamental steps and several reactive Pt sites are needed to promote the reaction.^73^ The MOR current (A) of the catalysts is normalized with the catalyst real surface area (*S*_r_) of Pt and total mass loaded of Pt to compare the specific and mass activities. The data of mass activities are given in Table 2, and their plots are given in Figure 6b and Figure S7. The mass-specific activities (*J*_m_) of the catalysts at their respective *E*_ap_ is in the order of Pt_1_Cu_1_ (0.47 A mg_Pt_^–1^) > Pt_1_Ni_1_ (0.41 A mg_Pt_^–1^) > Pt_1_Fe_1_ (0.29 A mg_Pt_^–1^) > Pt_1_Zn_1_ (0.27 A mg_Pt_^–1^) > Pt/C (0.26 A mg_Pt_^–1^) > Pt_1_Co_1_ (0.13 A mg_Pt_^–1^). The synthesized catalysts with Cu, Ni, Fe, and Zn have higher mass activities than the commercial Pt/C (20%) catalyst (Table 2 and Figure 6b).

**Figure 7:**
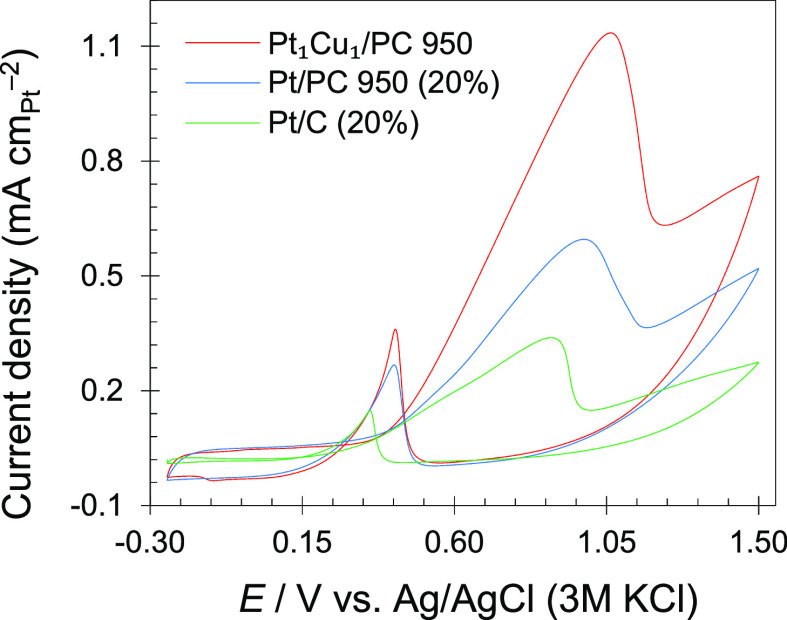
CV plots of the catalysts in HClO_4_ (0.1 mol L^–1^) and CH_3_OH (1 mol L^–1^) solution at 25 mV s^–1^.

To investigate the effect of the carbon support, the Pt 20% catalyst supported on PC 950 was electrochemically evaluated under similar experimental conditions and the results were compared to Pt_1_Cu_1_ on PC 950 and Pt 20% on commercial carbon (Figure 7). The Pt 20% catalyst on the PC 950 carbon support showed a higher current density compared to the catalyst of the commercial carbon Pt 20% catalyst. The high oxidation current observed with the coordination polymer-derived PC support revealed its importance in enhancing the MOR activities due to its porous structure that permit rapid molecular diffusion during reaction and boosted the methanol oxidation reaction. Therefore, besides the appropriate alloy composition of the synthesized catalyst, the porous morphology of the carbon support is also the main influencing factor for the improved MOR activities.

### Electrochemical Stability Tests and EIS Responses

**Figure 8:**
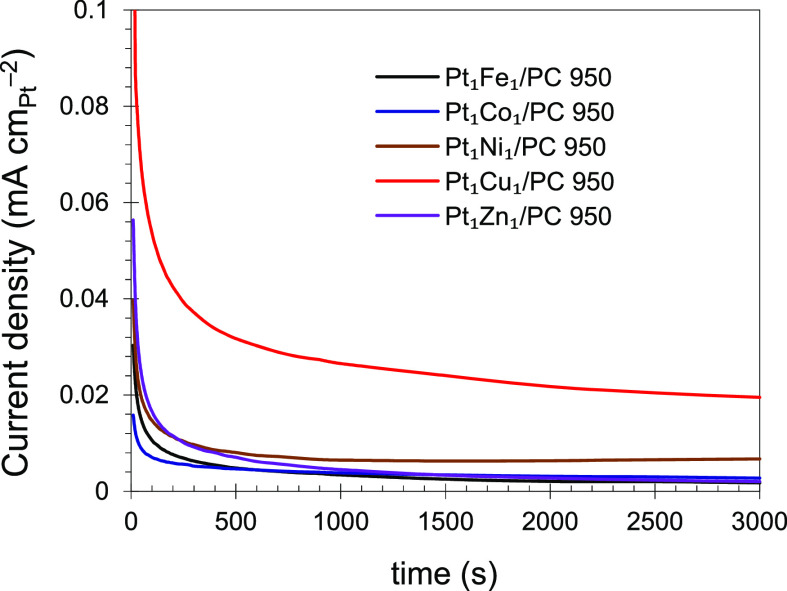
CA plots of the synthesized catalysts.

Chronoamperometry (CA) responses of the catalysts at +0.5 V were recorded for 3000 s, and the results are shown in Figure 8. Initially, the current decreased sharply with time (until 500 s) and then decreased slowly with time (until 3000 s). The decrease in current with respect to time is probably due to CO poisoning of PtM during methanol oxidation.^43^ In the case of the Pt_1_Cu_1_/PC 950 catalyst, the current decreased sharply for an initial 500 s and then became stable, suggesting better tolerance of the catalyst. The synthesized electrocatalyst (Pt_1_Cu_1_/PC 950) was the most stable among the options within the period of the CA experiment. From Figure 8, the stability order of the synthesized catalyst can be given as Pt_1_Cu_1_/PC 950 > Pt_1_Ni_1_/PC 950 > Pt_1_Zn_1_/PC 950 > Pt_1_Fe_1_/PC 950 > Pt_1_Co_1_/PC 950. This means that the PtM catalysts with M = Fe, Zn, and Co were deactivated in an acid solution when polarized at +0.5 V for 3000 s. However, the PtNi-based catalyst is also more stable as it not only showed a higher initial current but also maintained a comparatively higher current than that of PtFe, PtZn, and PtCo combinations in the completely experimental time.

The MOR responses of the catalysts were checked through CV after 3000 s of CA experiment, and the results are shown in Figure S8. A small decrease in *I*_ap_ was observed for PtCu (from 1.14 to 1.11 mA cm_Pt_^–2^) and PtNi (from 0.76 to 0.75 mA cm_Pt_^–2^); a moderate decrease in *I*_ap_ was observed for PtFe (from 0.57 to 0.54 mA cm_Pt_^–2^) and PtZn (from 0.51 to 0.49 mA cm_Pt_^–2^); and a significant decrease in *I*_ap_ was observed for PtCo (from 0.32 to 0.28 mA cm_Pt_^–2^) (Table S3). However, the overall trend of the catalyst (after CA) toward MOR remained the same (Figure S8f) and showed better performance than the initial CV results of Pt/C (20%). The decrease in current density of the catalysts after CA can be attributed to the NP CO-poisoning.

**Figure 9:**
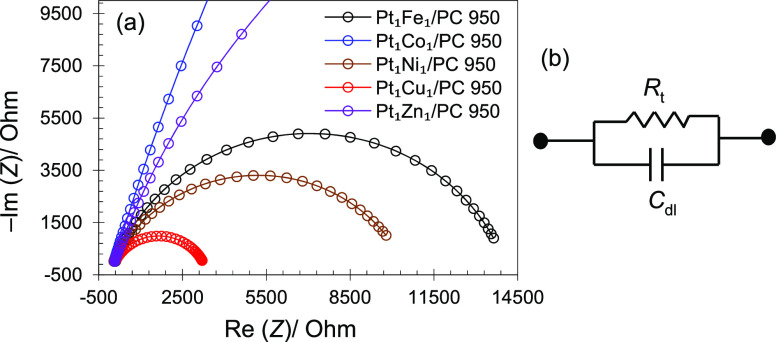
Nyquist plots of the catalysts (a) and equivalent circuit model (b). *C*_dl_ represent double-layer capacitance.

Electrochemical impedance spectroscopy (EIS) in the range from 1 Hz to 100 kHz at +0.5 V keeping the alternating current (AC) amplitude at 10 mV was carried out to check the interphase catalyst resistance. The Nyquist plots (Figure 9a) indicate that the PtM (M = Fe, Co, Ni, Cu, Zn) catalysts have different impedance behaviors toward MOR. Here, we can assume eqs 4 and 6 (methanol and CO oxidations) as a two-step model of MOR on PtM catalysts to make the mechanism simple. According to the reaction mechanism of MOR (eqs 4 and 6), methanol is adsorbed on Pt active sites, while OH comes from the dissociation of H_2_O on M sites. If *R*_1_ is the rate of methanol (eq 4) and *R*_2_ is the rate of CO (eq 6) oxidations, then *I* will be the net Faraday current generated (*I*_F_ = *R*_1_ + *R*_2_). According to the kinetic theory of MOR on the surface of electrode involving adsorption,^74^ the Faradaic current varies with the electrode potential (*E*) and with the fractional surface coverage of CO_ads_. Based on MOR and CO_ads_, the current flow (Faradic admittance, *Y*_F_) is given as^74^
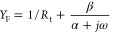
7where *R*_t_ is the charge transfer resistance (Ohm) of the electrode reaction and ω is the radial frequency (Hz) while other parameters such as β*,* α, and *j* are the parts of simulated equivalent circuit model defined in ref (74). Equation 7 shows that when the catalyst surface covered by CO_ads_ is kept constant, the *R*_t_ describing the kinetics of charge transfer changes with potential during MOR studies. Charge transfer resistance can also be define as *R*_t_ = lim_ω → 0_Re[*Z*_f_] where Re[*Z*_f_] is the real part of resistance and can be obtained from the Nyquist plots. Figure 9 shows that the diameter of the semi-circle (*R*_t_ part in the Figure 9b) increases for Pt with M in the order as Cu < Ni < Fe < Zn < Co, which is in good agreement with MOR electrochemical results. The smaller charge transfer resistance shown by the Pt_1_Cu_1_/PC 950 catalyst is due to the metals’ appropriate combination in alloy, uniform particle size distribution on support surface, and comparatively more stable and CO-tolerant behavior. The impedance analysis clearly demonstrates that the current density varies (at constant potential) while changing the catalyst composition.

## Conclusions

PtM (M = Fe, Co, Ni, Cu, Zn) binary electrocatalysts supported on PC 950, which is obtained by inert atmosphere thermal decomposition of a zinc-coordination polymer, were prepared and evaluated for MOR in an acid solution. In the electrochemical investigation, Pt_1_Cu_1_/PC 950 and Pt_1_Ni_1_/PC 950 catalysts exhibited more enhanced specific and mass activities than Pt_1_Fe_1_/PC 950, Pt_1_Zn_1_/PC 950, Pt_1_Co_1_/PC 950, Pt/PC 950, and Pt/C (20%). The Pt_1_Zn_1_/PC 950 and Pt/C (20%) catalysts showed comparable specific and mass activities, while Pt_1_Co_1_/PC showed the worst catalytic activity among all the prepared electrocatalysts. The high performance of PtCu and PtNi and the comparative performance of PtZn in low-pH MOR can be attributed to the change in the electronic properties of Pt and its significant effect on methanol chemisorption and oxidation. The porous morphology of the PC 950 has also boosted the MOR specific activities.

## Experimental Section

### Synthesis

The coordination polymer was synthesized by the reaction of zinc acetate dihydrate [Zn(O_2_CCH_3_)_2_·2H_2_O] and benzene-1,4-dicarboxylic acid [BDC; C_8_H_6_O_4_] according to our previously reported method.^9^ The PC was obtained by carbonizing a vacuum-dried zinc-coordination polymer at 950 °C in a tube furnace under argon. Details of polyol reduction method of catalysts synthesis is given in the Supporting Information.^8,13^ The salt precursor quantities for the synthesis of catalysts were used as follows: Pt_1_Cu_1_/PC 950 (Cu(NO_3_)_2_·3H_2_O salt of 142.6 mg; 0.590 mmol for 15% Cu), Pt_1_Fe_1_/PC 950 (FeCl_3_ salt of 108.63 mg; 0.670 mmol for 15% of Fe), Pt_1_Co_1_/PC 950 (Co(NO_3_)_2_·6H_2_O salt of 184.70 mg; 0.635 mmol for 15% of Co), Pt_1_Ni_1_/PC 950 (Ni(NO_3_)_2_·6H_2_O salt of 185.30 mg; 0.637 mmol for 15% of Ni), and Pt_1_Zn_1_/PC 950 (Zn(O_2_CCH_3_)_2_·2H_2_O salt of 125.57 mg; 0.572 mmol for 15% of Zn). For the Pt/PC 950 (20%) catalyst, 200 mg of carbon (80%) and 132 mg of H_2_PtCl_6_.6H_2_O (0.254 mmol for 20% of Pt) were used in the synthesis procedure. In the catalyst formula (Pt_1_M_1_/PC 950), the subscript digit is the equivalent metal loading in percent and 950 is the carbonization temperature.

### Characterization

Details of the instrumental characterization are available in the Supporting Information.

### Electrochemical Measurements

The catalyst ink was prepared by mixing 3 mg of each catalyst with 20 μL of Nafion 117 (5 wt %) and was diluted to 3 mL of 2-propanol. To make a homogeneous slurry, the mixture was sonicated for 1 h. Glassy carbon electrode was polished with alumina (0.5 μm). The sonicated ink of the catalyst (∼10 μL) was casted on a glassy carbon electrode surface (surface area = 0.247 cm^2^). For Pt_1_Ni_1_/ PC 950 and Pt_1_Cu_1_/PC 950, the Pt content on the working electrode was 60.73 μg cm^–2^, while for Pt_1_Fe_1_/ PC 950, Pt_1_Co_1_/ PC 950, Pt_1_Zn_1_/PC 950, Pt/PC 950, and Pt/C (20%) the Pt content on the electrode surface was 80.97 μg cm^–2^.^75^ Fresh ink was casted for every new experiment. The experiments were performed using a Biologic *SP-300* workstation. A three-electrode cell assembly, including glassy carbon casted with catalyst paste as working, Ag/AgCl (3.0 mol L^–1^ KCl) as reference, and Pt wire as counter electrodes, was used for cyclic voltammetry (CV) and linear sweep voltammetry (LSV). The electrolyte used was HClO_4_/CH_3_OH (0.1 mol L^–1^/1 mol L^–1^) solution. The voltammetry was run in the potential window from −0.25 to 1.5 V and at 25 mV s^–1^. The cell was purged with N_2_ (99.9% pure) for 5 min before experiment. The chronoamperometry experiments were performed at +0.5 V versus Ag/AgCl for 3000 s. For specific and mass activities, the CV plots of MOR were normalized with the catalyst real surface area (*S*_r_) and with Pt content loaded on the electrode surface.

## Supporting Information Available

Experimental procedures of catalysts synthesis, PXRD, XPS, TEM, and gas adsorption analysis; results of PXRD, XPS, and CV; and data of current density before and after CA tests (PDF)
