# Supplementary material for: Zinc-Coordination Polymer-Derived Porous Carbon-Supported Stable PtM Electrocatalysts for Methanol Oxidation Reaction
Source: ACS Omega. 2021 Mar 2;6(10):6780–90. doi: 10.1021/acsomega.0c05843 (PMC7970476; doi:10.1021/acsomega.0c05843)
Supplement: Supplementary file 1 — ao0c05843_si_001.pdf [file ao0c05843_si_001.pdf]

# Supporting Information

# Zinc-Coordination Polymer-Derived Porous Carbon-Supported Stable PtM Electrocatalysts for Methanol Oxidation Reaction

Inayat Ali Khan[1,2]*, Amin Badshah[1], Faiz Ullah Shah[2], Mohammed A. Assiri[3], and Muhammad Arif Nadeem[1]*

[1]*Catalysis and Nanomaterials Lab 27, Department of Chemistry, Quaid-i-Azam University, Islamabad 45320, Pakistan*

[2]*Chemistry of Interfaces, Luleå University of Technology, SE-97187 Luleå, Sweden*

[3]*Department of Chemistry, Faculty of Science, King Khalid University, P.O. Box 9004, Abha 61413, Saudi Arabia*

*Corresponding:

Email: inayat.khan@ltu.se, Phone: +46 (0) 920491738 (Dr. I.A. Khan)

Email: manadeem@qau.edu.pk, Phone: 092 051 90642062 (Dr. M.A. Nadeem)

Total Pages: 09
Total Figures: 08
Total Tables: 03

## EXPERIMENTAL

**Synthesis of Catalysts.** Polyol method was used for the synthesis of catalysts [230 mg each (30% PtM loading)]. In the synthesis of $Pt_1Cu_1$/PC 950, 175 mg of carbon support (70% of the total catalyst) was suspended in 20 mL of ethylene glycol using sonicator for 30 min and the suspension was kept at 100 °C under gentle stirring. Solutions of $H_2PtCl_6.6H_2O$ (99.55 mg; 0.192 mmol for 15% Pt) and $Cu(NO_3)_2.3H_2O$ (142.6 mg; 0.590 mmol for 15% Cu) were separately prepared in 10 mL ethylene glycol. The solutions were added to the carbon suspension dropwise and the temperature of the reaction mixture was gradually increased to 180 °C (2 °C $min^{-1}$). The mixture was reflux for 4 h under stirring. After reaction completion, the product was cooled to room temperature and was collected after filtration, washing and drying.

**Characterization.** X–ray diffractometer, RIGAKU MiniFlex600 with Cu $K_\alpha$ ($\lambda$ = 1.544206 Å) radiation at 40 kV and 15 mA and at scan of 0.02 deg $s^{-1}$, was used for powder X-ray (PXRD) analysis. Mercury 3.6 software was used to extract the simulated PXRD pattern of MOF-5. Kratos AXIS Ultra DLD instrument with vacuum better than $2\times10^{-9}$ mbar was used for XPS analysis. Radiation source of Al $K_\alpha$ (energy hν = 1486.68 eV), power of 164 W (15.2 kV and 10.8 mA) and spot size of 500 µm were used during measurements. The emitted photons was at 90° to the detector and adventitious carbon C 1s (284.5 eV) was used as reference and peaks in the spectrum were shifted accordingly. CasaXPS software was used for spectrum fitting with Shirley/Linear type background correction and asymmetric Gaussian/Lorentzians (0-30% Lorentzian character) was adopted. The FWHM in the range of 0.7-2 eV was maintained for sub-peaks, keeping instrumental specific parameters. In this way the peaks were added and the best fit was obtained. Gas adsorption analysis were carried out using Autosorb iQ3 gas sorption analyzer (Quantachrome Instruments USA). Prior to measurements each sample were degassed at 200 °C for 12 h under vacuum. A liquid nitrogen bath at 77 K was used for isotherms measurement. Bruner-Emmett-Teller (BET) model was used to calculate the surface area and the DFT method was used to calculate the porosity. JEOL-JEM 2010F FE-TEM field-emission transmission electron microscope at operating voltage of 200 kV was used for TEM analysis. For elemental analysis ICP spectrometer ICAP 6000 Series and CHNS analyzer 2400 Series II CHNS/O were used.

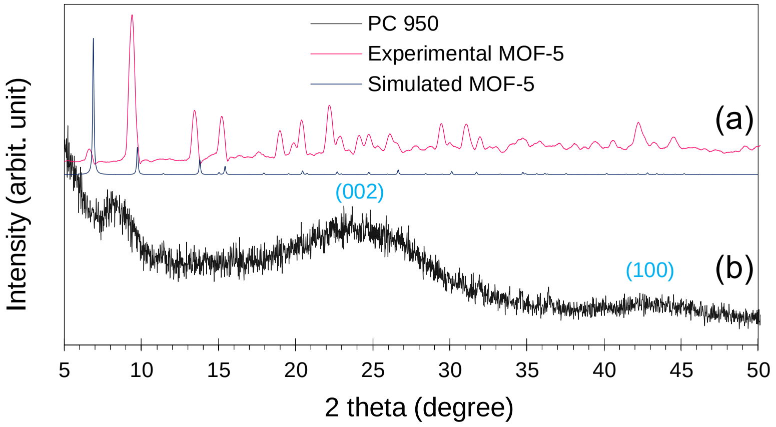


**Figure S1.** Simulated and experimental PXRD patterns of MOF-5 (a) and PXRD pattern of PC 950 (b).

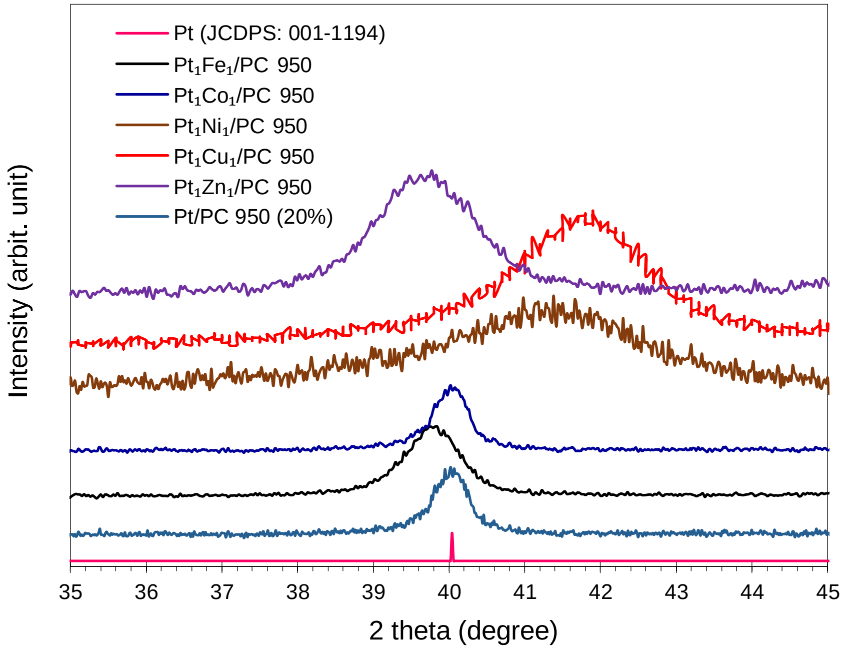


**Figure S2.** PXRD patterns of the synthesized catalysts from $2\theta$ 35° to $2\theta$ 45°.

**Table S1.** Crystallite sizes from PXRD, particle sizes from TEM, calculated and experimental compositions of the catalysts

| Catalysts | $\theta_{111}$ | $d_{111}$ (nm) | LP (nm) | FWHM (radian) | $D_{cryst}$ (nm) | TEM (nm) | at. %[a] | at. %[b] | Wt.% from ICP-OES | | |
|---|---|---|---|---|---|---|---|---|---|---|---|
| | | | | | | | | | Pt | M | Total |
| Pt/PC 950 (20%) | 20.09 | 0.224 | 0.388 | 1.063 | 7.985 | 4.7 | $Pt_{18}$ | - | 17.96 | - | 17.96 |
| $Pt_1Fe_1$//PC 950 | 20.00 | 0.226 | 0.390 | 0.015 | 09.83 | 9.410 | - | $Pt_{15}Fe_{07}$ | - | - | - |
| $Pt_1Co_1$//PC 950 | 20.03 | 0.225 | 0.400 | 0.007 | 24.00 | - | - | $Pt_{14}Co_{05}$ | - | - | - |
| $Pt_1Ni_1$//PC 950 | 20.85 | 0.217 | 0.375 | 0.035 | 04.00 | 3.00 | $Pt_{15}Ni_{11}$ | $Pt_{15}Ni_{12}$ | 15.08 | 11.06 | 26.14 |
| $Pt_1Cu_1$//PC 950 | 21.09 | 0.214 | 0.371 | 0.033 | 04.52 | 3.20 | $Pt_{13}Cu_{12}$ | $Pt_{13}Cu_{11}$ | 13.12 | 12.01 | 25.13 |
| $Pt_1Zn_1$//PC 950 | 19.77 | 0.229 | 0.389 | 0.014 | 10.61 | - | - | $Pt_{16}Zn_{10}$ | - | - | - |

[a]Calculated from ICP-OES (ICP-OES analysis was not performed for $Pt_1Fe_1$, $Pt_1Co_1$ and $Pt_1Zn_1$)
[b]Calculated from Vegard's law

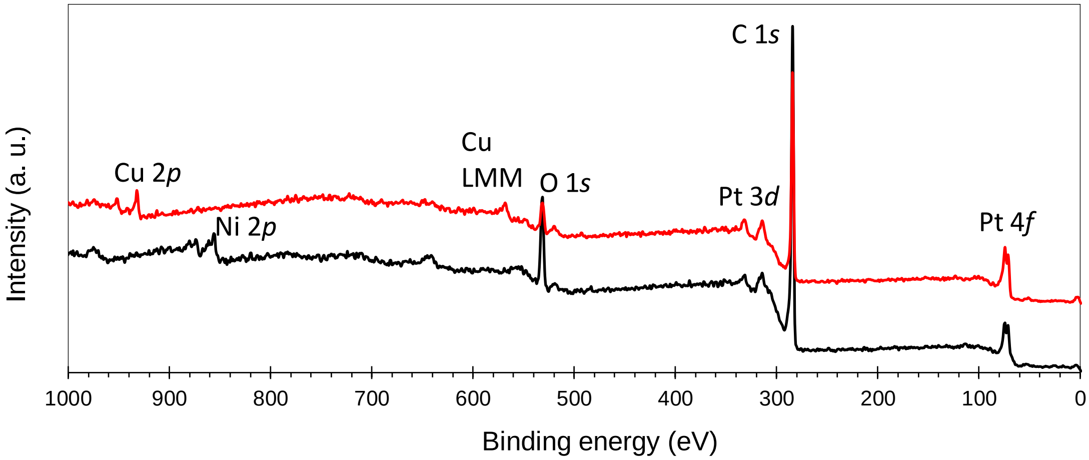


**Figure S3.** XPS spectra of the synthesized catalysts $Pt_1Ni_1$/PC 950 (black) and $Pt_1Cu_1$/PC 950 (red).

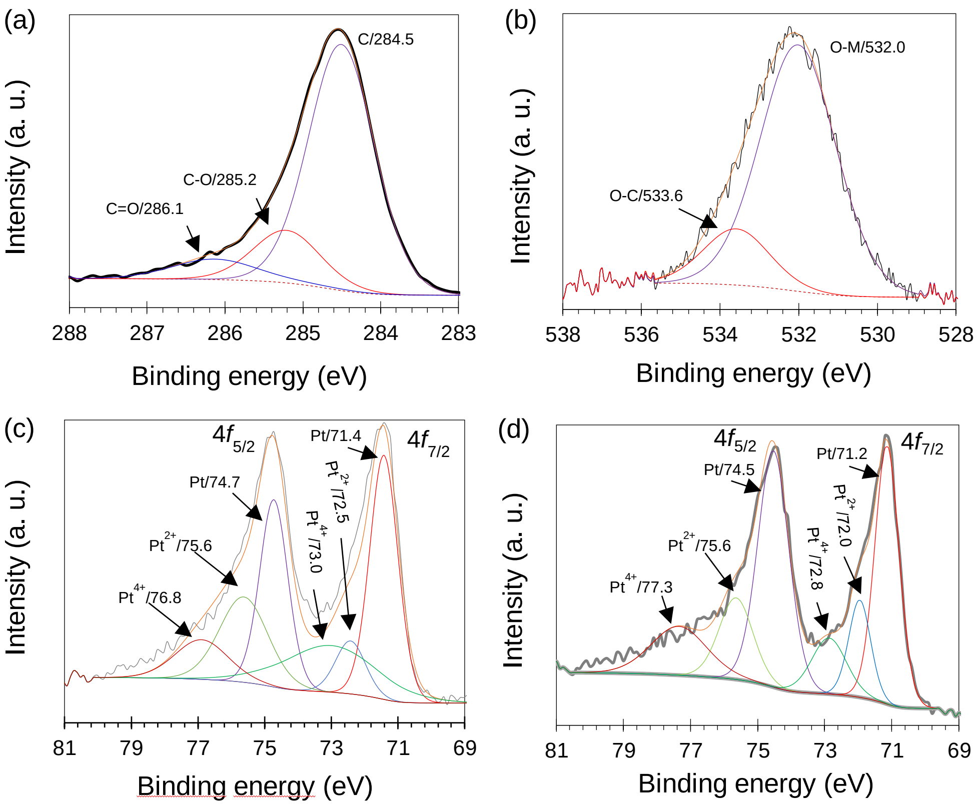


**Figure S4.** Representative deconvoluted XPS spectra of C 1*s* core line (a) and O 1*s* core line (b) in $Pt_1Ni_1$/PC 950. The deconvoluted XPS spectra of Pt 4*f* core line in PtNi (c) and Pt 4*f* core line in PtCu (d).

**Table S2**. Elemental wt.% quantity of the catalyst from XPS measurements

| Catalysts | Elements | Wt.% |
|---|---|---|
| $Pt_1Ni_1$/PC 950 | C 1*s* | 75.17 |
| | O 1*s* | 7.51 |
| | Pt $4f_{7/2}$ Pt $4f_{5/2}$ | 11.01 |
| | Ni $2p_{3/2}$ Ni $2p_{1/2}$ | 4.21 |
| $Pt_1Cu_1$/PC 950 | C 1*s* | 84.83 |
| | O 1*s* | 6.33 |
| | Pt $4f_{7/2}$ Pt $4f_{5/2}$ | 4.31 |
| | Cu $2p_{3/2}$ Cu $2p_{1/2}$ | 3.74 |

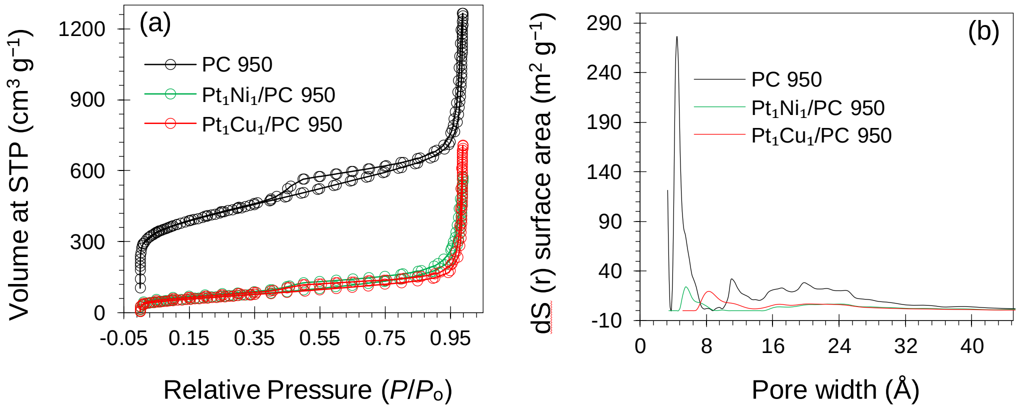


**Figure S5.** $N_2$-adsorption/desorption isotherms (a) and plots of pore size distribution (b).

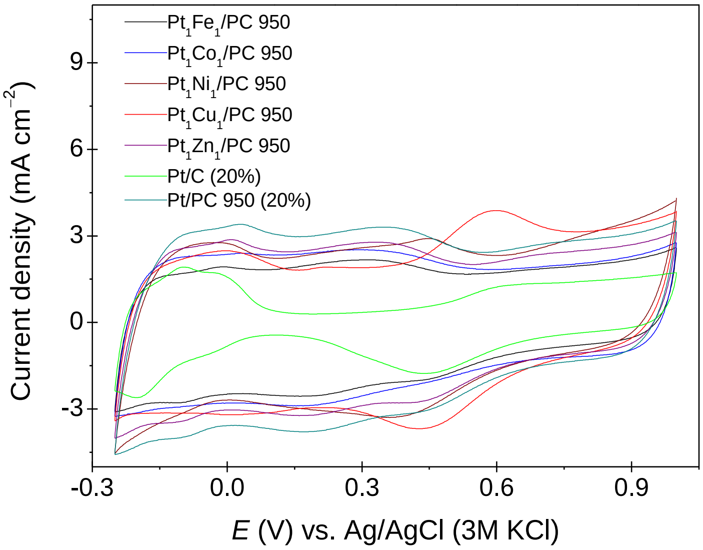


**Figure S6.** CV plots of the catalysts in $HClO_4$ (0.1 mol $L^{-1}$) solution and at 25 mV $s^{-1}$. The current was normalized with the geometrical surface area of electrode.

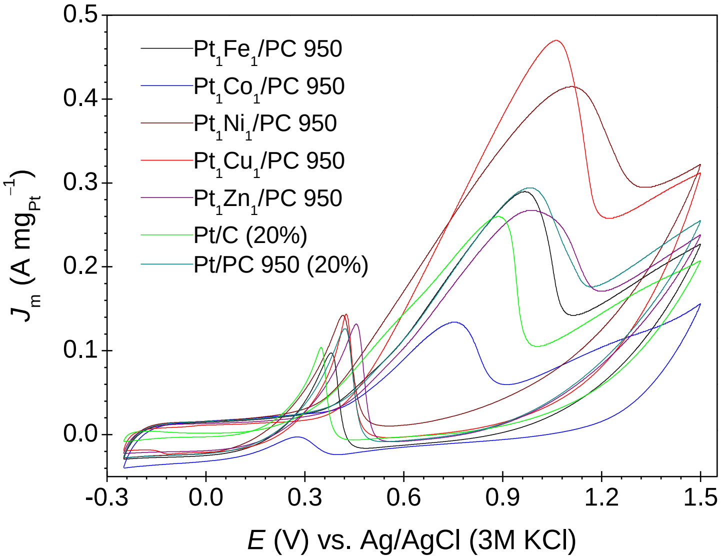


**Figure S7.** CV plots of the catalysts in $HClO_4$ (0.1 mol $L^{-1}$) and $CH_3OH$ (1 mol $L^{-1}$) solutions and at 25 mV $s^{-1}$.

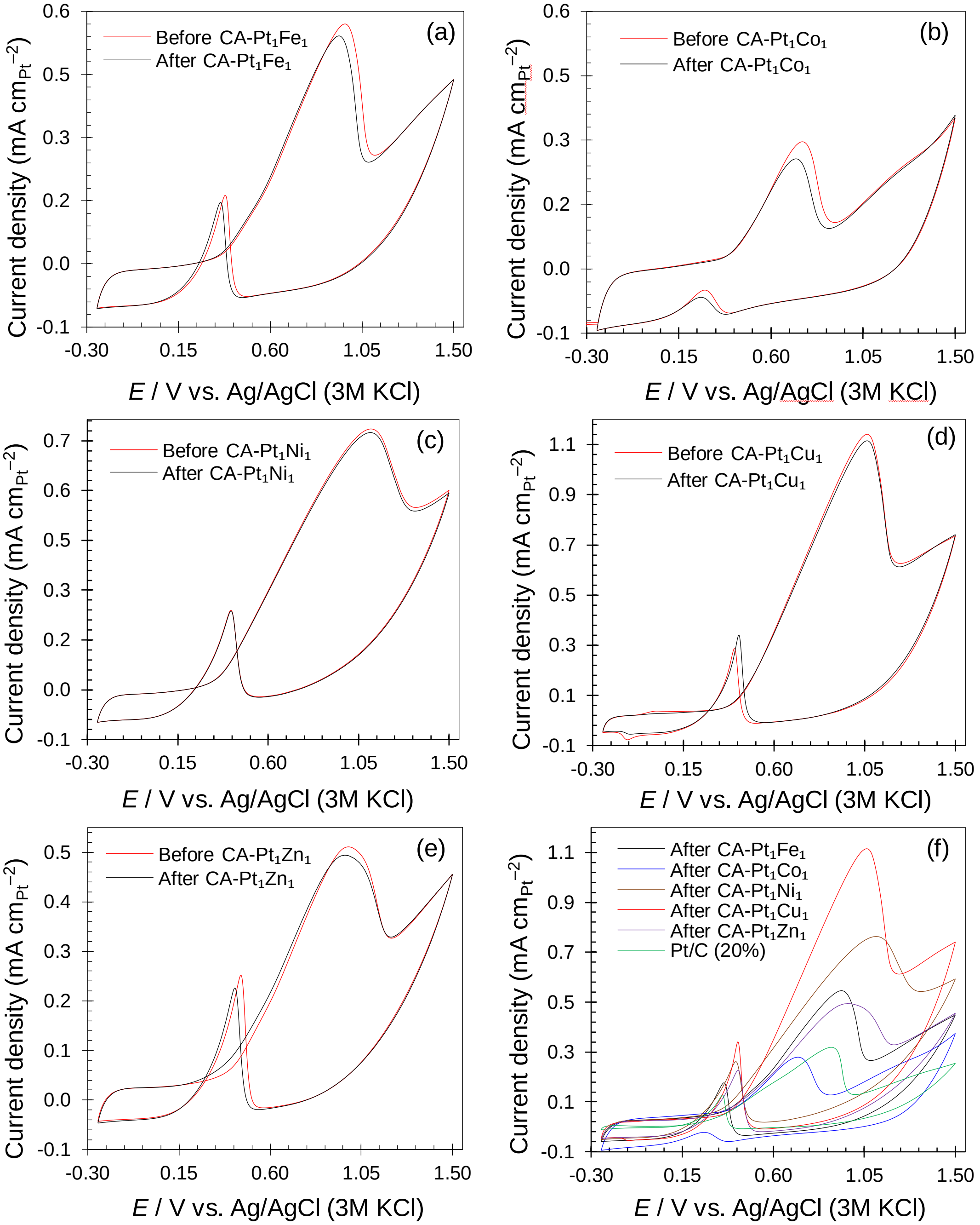


**Figure S8.** Comparative CV plots of the catalysts (a-e) and CV curves of the catalysts (PtM) after CA (f). CA experiment is not performed with Pt/C 20% catalyst.

**Table S3.** Comparative current densities of the PC 950 supported catalysts

| Catalysts | Before CA $I_{ap}$ (mA $cm_{Pt}^{-2}$) | After CA $I_{ap}$ (mA $cm_{Pt}^{-2}$) |
|---|---|---|
| $Pt_1Fe_1$ | 0.57 | 0.54 |
| $Pt_1Co_1$ | 0.32 | 0.28 |
| $Pt_1Ni_1$ | 0.76 | 0.75 |
| $Pt_1Cu_1$ | 1.14 | 1.11 |
| $Pt_1Zn_1$ | 0.51 | 0.50 |
